# *trans*-Di­chlorido­bis­(secnidazole-κ*N* ^3^)copper(II)

**DOI:** 10.1107/S2414314624003766

**Published:** 2024-05-03

**Authors:** Ismael Angel-Nieto, Rosa Elena Arroyo-Carmona, Aarón Pérez-Benítez, Gerardo Aguirre-Hernández, Sylvain Bernès

**Affiliations:** a Instituto de Física, Benemérita Universidad Autónoma de Puebla, Av. San Claudio y 18 Sur, 72570 Puebla, Pue., Mexico; b Facultad de Ciencias Químicas, Benemérita Universidad Autónoma de Puebla, Ciudad Universitaria, 72570 Puebla, Pue., Mexico; c Tecnológico Nacional de México, Instituto Tecnológico de Tijuana, Centro de Graduados e Investigación en Química, 22444 Tijuana BC, Mexico; University of Antofagasta, Chile

**Keywords:** crystal structure, coordination compound, secnidazole, supra­molecular structure, hydrogen bonds

## Abstract

The use of acetic acid (HOAc) in a reaction between CuCl_2_·2H_2_O and secnid­azole, an active pharmaceutical ingredient useful in the treatment against a variety of anaerobic Gram-positive and Gram-negative bacteria, affords the title complex, [CuCl_2_(C_7_H_11_N_3_O_3_)_2_]. This compound was previously synthesized using ethanol as solvent, although its crystal structure was not reported [Betanzos-Lara *et al.* (2013 ▸). *Inorg. Chim. Acta*, **397**, 94–100]. In the mol­ecular complex, the Cu^2+^ cation is situated at an inversion centre and displays a square-planar coordination environment. There is a hydrogen-bonded framework based on inter­molecular O—H⋯Cl inter­actions, characterized by H⋯Cl separations of 2.28 (4) Å and O—H⋯Cl angles of 175 (3)°. The resulting supra­molecular network is based on *R_2_^2^* (18) ring motifs, forming chains in the [010] direction.

## Abstract 2

The crystal structure of the title complex is stabilized by inter­molecular O—H⋯Cl hydrogen bonds, forming 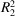 (18) ring motifs.

## Graphical abstract

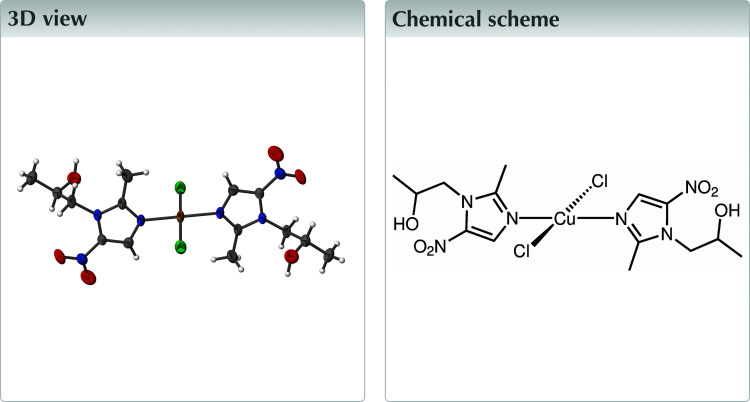

## Structure description

Secnidazole [C_7_H_11_N_3_O_3_, IUPAC name: 1-(2-methyl-5-nitro-1*H*-imidazol-1-yl)propan-2-ol, abbreviated *secnim*] is an active pharmaceutical ingredient used in the treatment against a variety of anaerobic Gram-positive and Gram-negative bacteria (Gillis & Wiseman, 1996 ▸). Some coordination complexes including secnidazole as a ligand were synthesized with late transition metals, Co^2+^, Ni^2+^, Cu^2+^ and Zn^2+^ (Betanzos-Lara *et al.*, 2013 ▸). Following the ideas of that group, the aim of this study is to obtain new complexes, to evaluate the synergistic effect of coordination of secnidazole to copper(II) on the anti­microbial activity.

In the literature, only one crystal structure of a secnidazole metallic complex has been reported (CSD refcode KICFUZ; Betanzos-Lara *et al.*, 2013 ▸). The complex consists of a dinuclear cluster of Cu^2+^ surrounded by four acetate anions OAc^−^ and two secnidazole mol­ecules bonded in terminal positions, to give [Cu_2_(*secnim*)_2_(μ_2_-OAc)_4_]. The same authors synthesized [Cu(*secnim*)_2_Cl_2_], although they did not determine its crystal structure. We have now obtained the same mononuclear complex using a simple synthetic route (see *Experimental*), and determined its mol­ecular and crystal structure.

**Figure 1:**
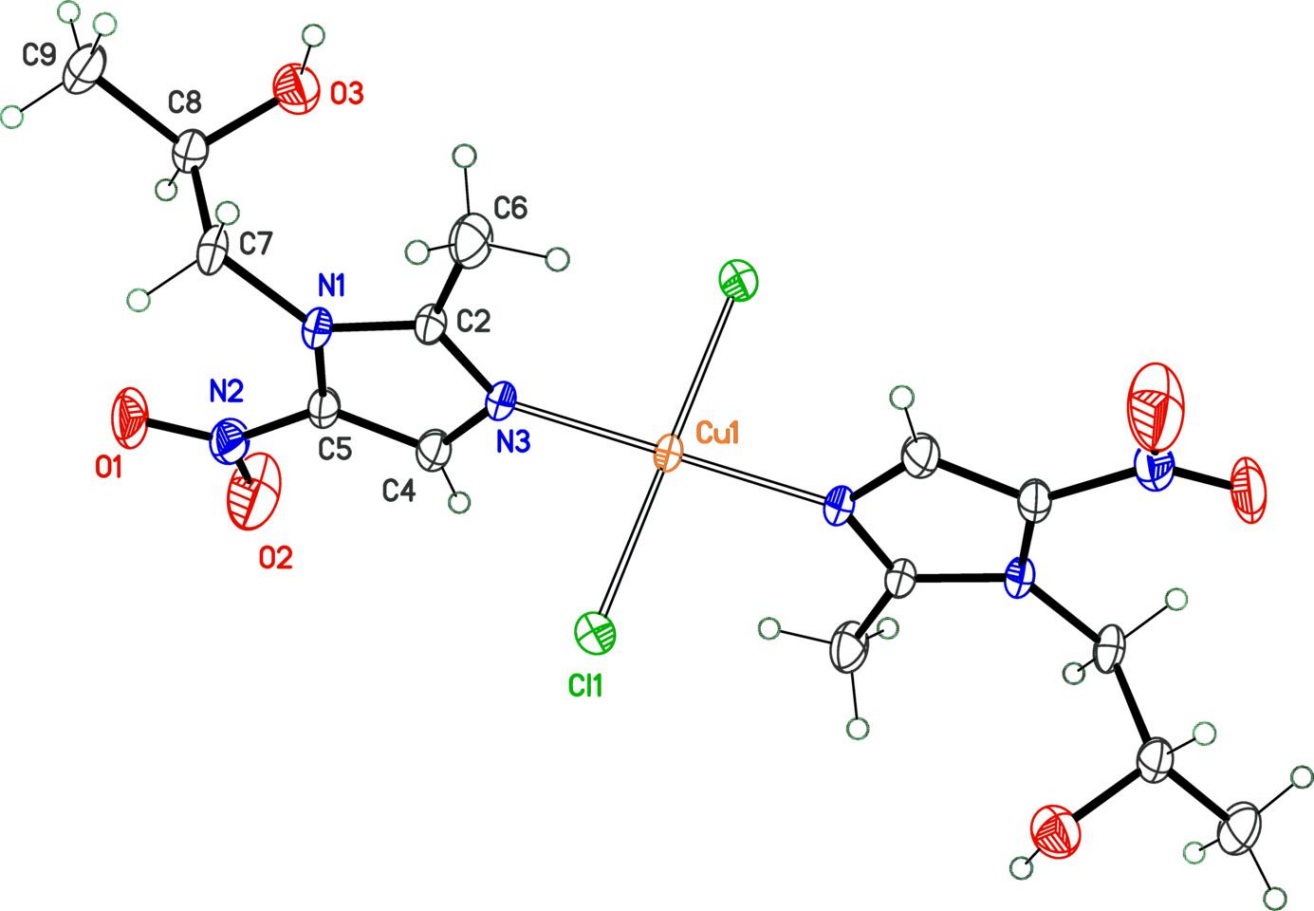
Mol­ecular structure of the title compound, with displacement ellipsoids for non-H atoms at the 30% probability level. Non-labelled atoms are generated by the symmetry operation 1 − *x*, −*y*, 1 − *z*.

**Figure 2:**
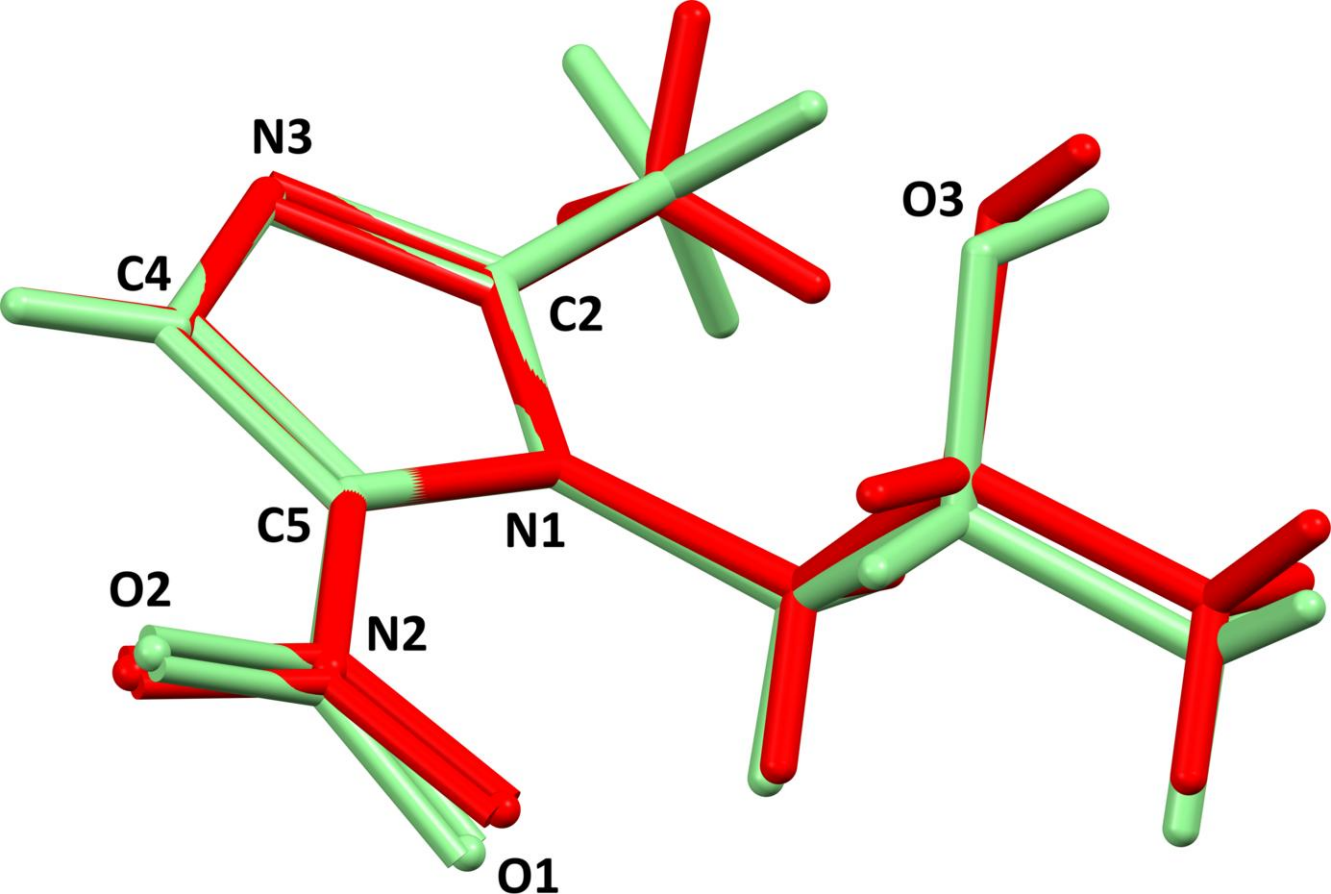
An overlay calculated with *Mercury* (Macrae *et al.*, 2020 ▸), comparing the shape of secnidazole as free ligand (red mol­ecule; Novoa de Armas *et al.*, 1997 ▸) and in the title compound (green mol­ecule). The overlay was computed using the five atoms belonging to the imidazole heterocycle. Note the small rotation of *ca* 14° for the nitro group.

The mononuclear Cu^2+^ ion is surrounded by two *secnim* mol­ecules *trans*-coordinated through the imidazolic nitro­gen atom N3, and two chloride ions, giving a distorted square-plane geometry for Cu^II^, with Cu1—N3 and Cu1—Cl1 bond lengths being 1.9953 (19) Å and 2.2586 (6) Å, respectively. The metal is located at an inversion centre in space group *P* 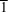 , and the asymmetric unit contains half a mol­ecule (Fig. 1 ▸). A mol­ecular overlay shows that the global conformation of the *secnim* free ligand (Novoa de Armas *et al.*, 1997 ▸) is not altered by coordination to the central metal (Fig. 2 ▸). The most significant modification is related to the free rotation of the NO_*2*_ group bonded to C5 in the ligand. The dihedral angle between the nitro group and the mean plane of the imidazole ring is 1.0° in the non-coordinating ligand, while this angle is 15.2 (4)° in the complex. Such a rotation could be a consequence of a steric hindrance between the nitro group and the propan-2-ol lateral chain in a neighbouring mol­ecule in the crystal (Table 1 ▸, entry 2).

**Figure 3:**
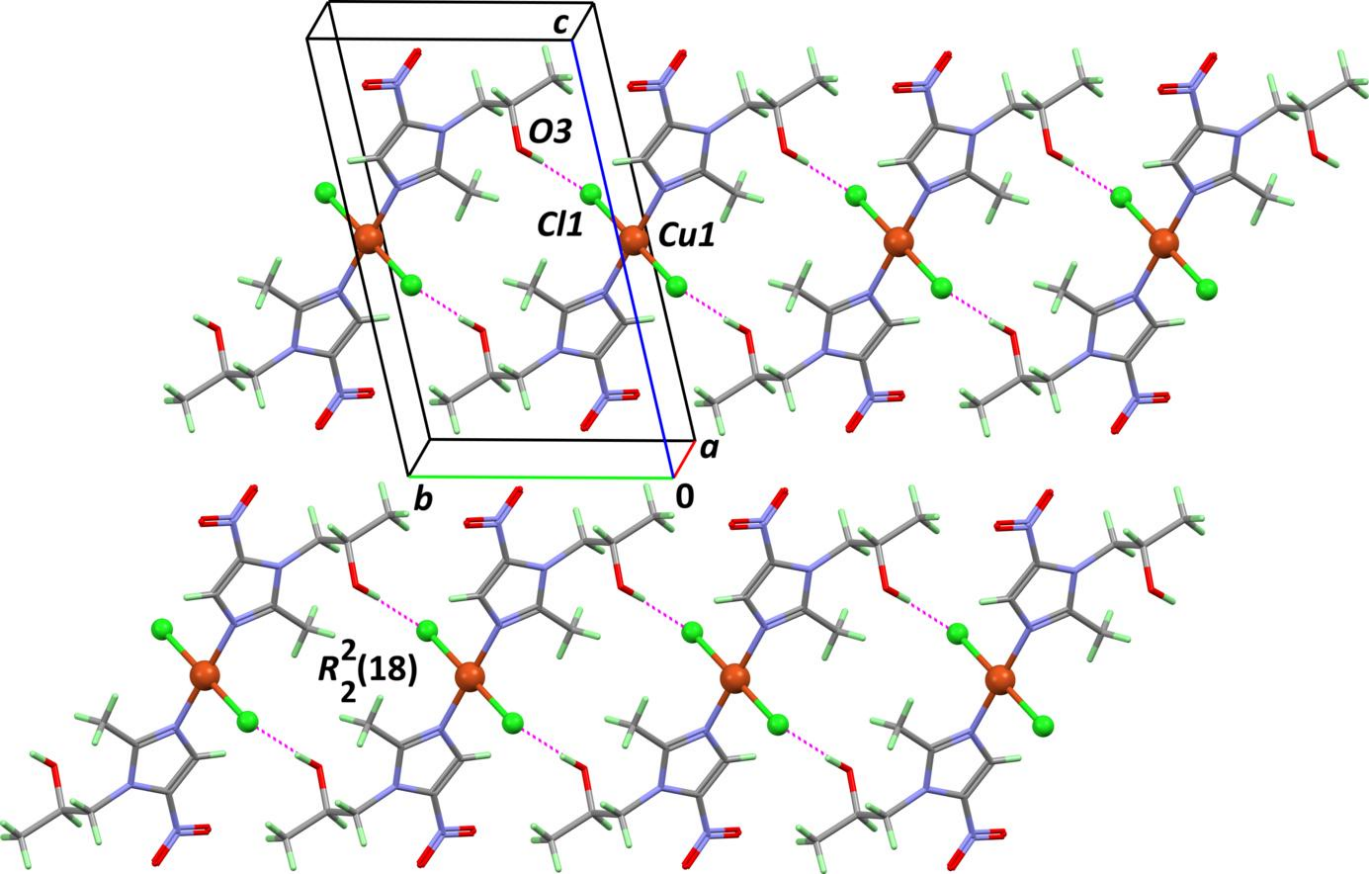
Part of the supra­molecular framework based on inter­molecular O—H⋯Cl hydrogen bonds (dashed purple lines) corresponding to the first entry in Table 1 ▸, as viewed down [100].

The orientation of the hy­droxy group promotes the formation of inter­molecular hydrogen bonds and acts as a donor to the chloride ion, which acts as an acceptor (Table 1 ▸, entry 1). The crystal structure features centrosymmetric 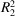 (18) ring motifs formed by the inter­action between the non-coordinating hydroxy group and the chloride ion of a symmetry-related complex. A periodic framework is created, based on chains running in the [010] direction (Fig. 3 ▸). These chains are parallel in the crystal, and inter­act poorly, through weak C—H⋯O contacts involving the hy­droxy and nitro groups as acceptors (Table 1 ▸, entries 2 and 3).

## Synthesis and crystallization

Two ethano­lic solutions of *secnim* (185 mg, 1 mmol in 15 ml) and CuCl_2_·2H_2_O (170 mg, 1 mmol, in 15 ml) were prepared at ambient conditions. Acetic acid (5 ml) was added to the CuCl_2_·2H_2_O solution. The solutions were combined under stirring for 1 h at 333 K. The resulting solution was then filtered and allowed to evaporate at 298 K over 2 days, affording blue single crystals suitable for X-ray crystallography.

## Refinement

Crystal data, data collection and structure refinement details are summarized in Table 2 ▸.

## Supplementary Material

Crystal structure: contains datablock(s) I. DOI: 10.1107/S2414314624003766/bx4028sup1.cif

Structure factors: contains datablock(s) I. DOI: 10.1107/S2414314624003766/bx4028Isup2.hkl

CCDC reference: 2350906

Additional supporting information:  crystallographic information; 3D view; checkCIF report

## Figures and Tables

**Table 1:** Hydrogen-bond geometry (Å, °)

*D*—H⋯*A*	*D*—H	H⋯*A*	*D*⋯*A*	*D*—H⋯*A*
O3—H3⋯Cl1^i^	0.97 (4)	2.28 (4)	3.252 (2)	175 (3)
C7—H7*A*⋯O3^ii^	0.97	2.40	3.327 (3)	160
C7—H7*B*⋯O1^iii^	0.97	2.51	3.436 (3)	159

Symmetry codes: (i) 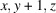 ; (ii) 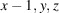 ; (iii) 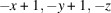 .

**Table 2:** Experimental details

Crystal data	
Chemical formula	[CuCl_2_(C_7_H_11_N_3_O_3_)_2_]
*M*_r_	504.81
Crystal system, space group	Triclinic, *P* 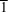
Temperature (K)	298
*a*, *b*, *c* (Å)	4.6536 (3), 8.2542 (3), 13.6820 (6)
α, β, γ (°)	78.092 (4), 82.801 (4), 85.469 (4)
*V* (Å^3^)	509.42 (4)
*Z*	1
Radiation type	Mo *K*α
μ (mm^−1^)	1.38
Crystal size (mm)	0.28 × 0.21 × 0.10

Data collection	
Diffractometer	SuperNova, Dual, AtlasS2
Absorption correction	Multi-scan (*CrysAlis PRO*; Rigaku OD, 2022 ▸)
*T*_min_, *T*_max_	0.879, 1.000
No. of measured, independent and observed [*I* > 2σ(*I*)] reflections	11147, 2582, 1956
*R*_int_	0.066
(sin θ/λ)_max_ (Å^−1^)	0.692

Refinement	
*R*[*F* ^2^ > 2σ(*F* ^2^)], *wR*(*F* ^2^), *S*	0.041, 0.108, 1.05
No. of reflections	2582
No. of parameters	139
H-atom treatment	H atoms treated by a mixture of independent and constrained refinement
Δρ_max_, Δρ_min_ (e Å^−3^)	0.70, −0.33

Computer programs: *CrysAlis PRO* (Rigaku OD, 2022 ▸), *SHELXT2018/2* (Sheldrick, 2015*a* ▸), *SHELXL2019/2* (Sheldrick, 2015*b* ▸), *XP* in *SHELXTL-Plus* (Sheldrick, 2008 ▸), *Mercury* (Macrae *et al.*, 2020 ▸) and *publCIF* (Westrip, 2010 ▸).

## full crystallographic data

### Crystal data

**Table:**

[CuCl_2_(C_7_H_11_N_3_O_3_)_2_]	*Z* = 1
*M**_r_* = 504.81	*F*(000) = 259
Triclinic, *P*1	*D*_x_ = 1.646 Mg m^−^^3^
*a* = 4.6536 (3) Å	Mo *K*α radiation, λ = 0.71073 Å
*b* = 8.2542 (3) Å	Cell parameters from 4331 reflections
*c* = 13.6820 (6) Å	θ = 4.3–28.4°
α = 78.092 (4)°	µ = 1.38 mm^−^^1^
β = 82.801 (4)°	*T* = 298 K
γ = 85.469 (4)°	Plate, blue
*V* = 509.42 (4) Å^3^	0.28 × 0.21 × 0.10 mm

### Data collection

**Table:**

SuperNova, Dual, AtlasS2 diffractometer	2582 independent reflections
Radiation source: micro-focus sealed X-ray tube, SuperNova (Mo) X-ray Source	1956 reflections with *I* > 2σ(*I*)
Mirror monochromator	*R*_int_ = 0.066
Detector resolution: 5.1970 pixels mm^-1^	θ_max_ = 29.5°, θ_min_ = 3.2°
ω scans	*h* = −6→5
Absorption correction: multi-scan (CrysAlisPro; Rigaku OD, 2022)	*k* = −11→11
*T*_min_ = 0.879, *T*_max_ = 1.000	*l* = −18→18
11147 measured reflections	

### Refinement

**Table:**

Refinement on *F*^2^	Primary atom site location: dual
Least-squares matrix: full	Secondary atom site location: difference Fourier map
*R*[*F*^2^ > 2σ(*F*^2^)] = 0.041	Hydrogen site location: mixed
*wR*(*F*^2^) = 0.108	H atoms treated by a mixture of independent and constrained refinement
*S* = 1.05	*w* = 1/[σ^2^(*F*_o_^2^) + (0.0399*P*)^2^ + 0.261*P*] where *P* = (*F*_o_^2^ + 2*F*_c_^2^)/3
2582 reflections	(Δ/σ)_max_ < 0.001
139 parameters	Δρ_max_ = 0.70 e Å^−^^3^
0 restraints	Δρ_min_ = −0.33 e Å^−^^3^
0 constraints	

### Special details

**Table:**

Refinement. Methine, methylene and methyl H atoms were refined using a riding model and calculated displacement parameters, while hydroxy H atom (H3) was refined with free coordinates and isotropic displacement parameter.

### Fractional atomic coordinates and isotropic or equivalent isotropic displacement parameters (Å^2^)

**Table:**

	*x*	*y*	*z*	*U*_iso_*/*U*_eq_	
Cu1	0.500000	0.000000	0.500000	0.04075 (17)	
Cl1	0.26976 (17)	−0.14756 (8)	0.41467 (5)	0.0491 (2)	
O1	0.7606 (6)	0.3821 (3)	0.04775 (15)	0.0687 (7)	
O2	1.0650 (7)	0.1867 (4)	0.0998 (2)	0.1057 (12)	
O3	0.7513 (5)	0.6770 (3)	0.27030 (16)	0.0542 (5)	
N1	0.5217 (4)	0.3783 (2)	0.24801 (14)	0.0310 (4)	
N2	0.8580 (6)	0.2806 (3)	0.11389 (17)	0.0495 (6)	
N3	0.5786 (5)	0.1665 (2)	0.37287 (15)	0.0381 (5)	
C2	0.4408 (5)	0.3135 (3)	0.34601 (17)	0.0324 (5)	
C4	0.7580 (7)	0.1368 (3)	0.29152 (19)	0.0446 (7)	
H4	0.882134	0.043325	0.289156	0.054*	
C5	0.7251 (6)	0.2663 (3)	0.21463 (18)	0.0371 (6)	
C6	0.2311 (6)	0.3957 (4)	0.4130 (2)	0.0488 (7)	
H6A	0.308953	0.495324	0.422023	0.073*	
H6B	0.195815	0.322391	0.477062	0.073*	
H6C	0.052384	0.422977	0.383621	0.073*	
C7	0.4181 (5)	0.5424 (3)	0.19434 (19)	0.0367 (6)	
H7A	0.243775	0.579365	0.232279	0.044*	
H7B	0.367519	0.531933	0.129350	0.044*	
C8	0.6415 (6)	0.6722 (3)	0.17891 (19)	0.0408 (6)	
H8	0.804035	0.640327	0.132601	0.049*	
C9	0.5105 (8)	0.8384 (4)	0.1286 (2)	0.0617 (9)	
H9A	0.350851	0.873264	0.172485	0.093*	
H9B	0.442936	0.827624	0.066869	0.093*	
H9C	0.655143	0.919358	0.114551	0.093*	
H3	0.598 (9)	0.730 (5)	0.310 (3)	0.084 (12)*	

### Atomic displacement parameters (Å^2^)

**Table:**

	*U*^11^	*U*^22^	*U*^33^	*U*^12^	*U*^13^	*U*^23^
Cu1	0.0753 (4)	0.0250 (2)	0.0210 (2)	−0.0102 (2)	−0.0118 (2)	0.00433 (16)
Cl1	0.0764 (5)	0.0412 (4)	0.0320 (3)	−0.0149 (3)	−0.0117 (3)	−0.0050 (3)
O1	0.1028 (18)	0.0693 (15)	0.0242 (10)	0.0088 (13)	0.0001 (11)	0.0037 (10)
O2	0.133 (3)	0.096 (2)	0.0610 (17)	0.056 (2)	0.0316 (16)	−0.0013 (15)
O3	0.0585 (13)	0.0574 (13)	0.0476 (12)	0.0023 (10)	−0.0117 (10)	−0.0113 (10)
N1	0.0391 (11)	0.0283 (10)	0.0222 (10)	−0.0017 (8)	−0.0046 (8)	0.0037 (8)
N2	0.0718 (17)	0.0423 (13)	0.0300 (12)	0.0029 (12)	0.0036 (11)	−0.0051 (10)
N3	0.0633 (14)	0.0253 (10)	0.0229 (10)	−0.0046 (9)	−0.0065 (9)	0.0030 (8)
C2	0.0423 (13)	0.0289 (12)	0.0237 (11)	−0.0070 (10)	−0.0049 (9)	0.0024 (9)
C4	0.0704 (19)	0.0309 (13)	0.0300 (14)	0.0092 (12)	−0.0083 (12)	−0.0032 (11)
C5	0.0540 (15)	0.0312 (12)	0.0230 (12)	0.0018 (11)	−0.0028 (10)	−0.0006 (10)
C6	0.0539 (17)	0.0496 (16)	0.0347 (15)	−0.0017 (13)	0.0105 (12)	0.0007 (12)
C7	0.0405 (13)	0.0358 (13)	0.0273 (12)	0.0044 (10)	−0.0069 (10)	0.0082 (10)
C8	0.0559 (16)	0.0334 (13)	0.0297 (13)	0.0002 (12)	−0.0048 (11)	0.0006 (10)
C9	0.097 (3)	0.0335 (15)	0.0472 (18)	0.0074 (15)	−0.0099 (17)	0.0058 (13)

### Geometric parameters (Å, º)

**Table:**

Cu1—N3	1.9953 (19)	C2—C6	1.477 (4)
Cu1—N3^i^	1.9953 (19)	C4—C5	1.350 (3)
Cu1—Cl1	2.2586 (6)	C4—H4	0.9300
Cu1—Cl1^i^	2.2586 (6)	C6—H6A	0.9600
O1—N2	1.207 (3)	C6—H6B	0.9600
O2—N2	1.210 (3)	C6—H6C	0.9600
O3—C8	1.417 (3)	C7—C8	1.517 (4)
O3—H3	0.97 (4)	C7—H7A	0.9700
N1—C2	1.355 (3)	C7—H7B	0.9700
N1—C5	1.373 (3)	C8—C9	1.520 (4)
N1—C7	1.478 (3)	C8—H8	0.9800
N2—C5	1.424 (3)	C9—H9A	0.9600
N3—C2	1.331 (3)	C9—H9B	0.9600
N3—C4	1.358 (3)	C9—H9C	0.9600
			
N3—Cu1—N3^i^	180.0	C2—C6—H6A	109.5
N3—Cu1—Cl1	88.73 (6)	C2—C6—H6B	109.5
N3^i^—Cu1—Cl1	91.27 (6)	H6A—C6—H6B	109.5
N3—Cu1—Cl1^i^	91.27 (6)	C2—C6—H6C	109.5
N3^i^—Cu1—Cl1^i^	88.73 (6)	H6A—C6—H6C	109.5
Cl1—Cu1—Cl1^i^	180.00 (4)	H6B—C6—H6C	109.5
C8—O3—H3	106 (2)	N1—C7—C8	112.9 (2)
C2—N1—C5	105.98 (19)	N1—C7—H7A	109.0
C2—N1—C7	124.6 (2)	C8—C7—H7A	109.0
C5—N1—C7	129.3 (2)	N1—C7—H7B	109.0
O1—N2—O2	123.5 (3)	C8—C7—H7B	109.0
O1—N2—C5	119.7 (2)	H7A—C7—H7B	107.8
O2—N2—C5	116.8 (2)	O3—C8—C7	111.3 (2)
C2—N3—C4	107.4 (2)	O3—C8—C9	113.4 (2)
C2—N3—Cu1	127.62 (18)	C7—C8—C9	109.2 (2)
C4—N3—Cu1	124.32 (17)	O3—C8—H8	107.6
N3—C2—N1	110.2 (2)	C7—C8—H8	107.6
N3—C2—C6	125.2 (2)	C9—C8—H8	107.6
N1—C2—C6	124.6 (2)	C8—C9—H9A	109.5
C5—C4—N3	108.2 (2)	C8—C9—H9B	109.5
C5—C4—H4	125.9	H9A—C9—H9B	109.5
N3—C4—H4	125.9	C8—C9—H9C	109.5
C4—C5—N1	108.2 (2)	H9A—C9—H9C	109.5
C4—C5—N2	126.7 (2)	H9B—C9—H9C	109.5
N1—C5—N2	125.0 (2)		
			
C4—N3—C2—N1	1.5 (3)	C2—N1—C5—C4	1.2 (3)
Cu1—N3—C2—N1	−169.46 (16)	C7—N1—C5—C4	177.2 (2)
C4—N3—C2—C6	−178.1 (3)	C2—N1—C5—N2	176.6 (3)
Cu1—N3—C2—C6	10.9 (4)	C7—N1—C5—N2	−7.4 (4)
C5—N1—C2—N3	−1.7 (3)	O1—N2—C5—C4	162.4 (3)
C7—N1—C2—N3	−178.0 (2)	O2—N2—C5—C4	−16.9 (5)
C5—N1—C2—C6	177.9 (2)	O1—N2—C5—N1	−12.1 (4)
C7—N1—C2—C6	1.6 (4)	O2—N2—C5—N1	168.5 (3)
C2—N3—C4—C5	−0.8 (3)	C2—N1—C7—C8	103.5 (3)
Cu1—N3—C4—C5	170.60 (19)	C5—N1—C7—C8	−71.9 (3)
N3—C4—C5—N1	−0.3 (3)	N1—C7—C8—O3	−51.0 (3)
N3—C4—C5—N2	−175.6 (3)	N1—C7—C8—C9	−176.9 (2)

Symmetry code: (i) −*x*+1, −*y*, −*z*+1.

### Hydrogen-bond geometry (Å, º)

**Table:**

*D*—H···*A*	*D*—H	H···*A*	*D*···*A*	*D*—H···*A*
O3—H3···Cl1^ii^	0.97 (4)	2.28 (4)	3.252 (2)	175 (3)
C4—H4···Cl1^iii^	0.93	2.81	3.537 (3)	136
C6—H6*A*···Cl1^ii^	0.96	2.92	3.793 (3)	152
C6—H6*B*···Cl1^iv^	0.96	2.79	3.546 (3)	136
C7—H7*A*···O3^v^	0.97	2.40	3.327 (3)	160
C7—H7*B*···O1^vi^	0.97	2.51	3.436 (3)	159

Symmetry codes: (ii) *x*, *y*+1, *z*; (iii) *x*+1, *y*, *z*; (iv) −*x*, −*y*, −*z*+1; (v) *x*−1, *y*, *z*; (vi) −*x*+1, −*y*+1, −*z*.
